# DNA Vaccines Encoding Antigen Targeted to MHC Class II Induce Influenza-Specific CD8^+^ T Cell Responses, Enabling Faster Resolution of Influenza Disease

**DOI:** 10.3389/fimmu.2016.00321

**Published:** 2016-08-23

**Authors:** Laura Lambert, Ekaterina Kinnear, Jacqueline U. McDonald, Gunnveig Grodeland, Bjarne Bogen, Elisabeth Stubsrud, Mona M. Lindeberg, Agnete Brunsvik Fredriksen, John S. Tregoning

**Affiliations:** ^1^Mucosal Infection and Immunity Group, Section of Virology, Department of Medicine, St. Mary’s Campus, Imperial College London, London, UK; ^2^K. G. Jebsen Centre for Influenza Vaccine Research, Institute of Clinical Medicine, Oslo University Hospital, University of Oslo, Oslo, Norway; ^3^Centre for Immune Regulation, Institute for Immunology, Oslo University Hospital, University of Oslo, Oslo, Norway; ^4^Vaccibody AS, Oslo, Norway

**Keywords:** influenza vaccines, CD8^+^ T-lymphocytes, DNA vaccines, MHC II, immune targeting

## Abstract

Current influenza vaccines are effective but imperfect, failing to cover against emerging strains of virus and requiring seasonal administration to protect against new strains. A key step to improving influenza vaccines is to improve our understanding of vaccine-induced protection. While it is clear that antibodies play a protective role, vaccine-induced CD8^+^ T cells can improve protection. To further explore the role of CD8^+^ T cells, we used a DNA vaccine that encodes antigen dimerized to an immune cell targeting module. Immunizing CB6F1 mice with the DNA vaccine in a heterologous prime-boost regime with the seasonal protein vaccine improved the resolution of influenza disease compared with protein alone. This improved disease resolution was dependent on CD8^+^ T cells. However, DNA vaccine regimes that induced CD8^+^ T cells alone were not protective and did not boost the protection provided by protein. The MHC-targeting module used was an anti-I-E^d^ single chain antibody specific to the BALB/c strain of mice. To test the role of MHC targeting, we compared the response between BALB/c, C57BL/6 mice, and an F1 cross of the two strains (CB6F1). BALB/c mice were protected, C57BL/6 were not, and the F1 had an intermediate phenotype; showing that the targeting of antigen is important in the response. Based on these findings, and in agreement with other studies using different vaccines, we conclude that, in addition to antibody, inducing a protective CD8 response is important in future influenza vaccines.

## Introduction

The annual burden of influenza is significant, with the WHO estimating one billion cases of infection a year. Of these, an estimate from 2008 suggests that about 90 million cases are in children under 5 years of age (1). This huge burden of disease is in spite of there being seasonal vaccines for influenza: these vaccines are not available for the global population and, due to the changing nature of circulating influenza strains, are often not completely effective. Ideally, new vaccines with broader cross protection would be developed, which address the problem of antigenic drift and the narrow window in which a seasonal vaccine is effective.

For the current generation of strain-specific protein vaccines, antibody is a valuable correlate of protection. Currently vaccines are licensed based on a hemagglutination inhibition (HAI) titer of 1:40, a surrogate assay for neutralizing antibody. However, the HAI assay has limitations, even for assessing antibody: it only measures anti-hemagglutinin responses and does not recognize all hemagglutinin-specific antibodies, for example, it does not detect antibodies that bind the more conserved stem region. Therefore, for the next generation of influenza vaccines, particularly for cross-reactive vaccines, better understanding about the relative contributions of different arms of the adaptive immune system in protection is required (2). For example, HAI titer fails to take into account the role of T cells in the vaccine response to influenza, which may also contribute to cross protection (3).

While both CD4^+^ and CD8^+^ T cells can contribute to protection against influenza, CD8^+^ T cells are particularly beneficial when they recognize conserved epitopes across multiple influenza strains (4). The direct evidence for the role of influenza disease reduction by CD8^+^ T cells is mostly derived from animal studies, but a recent study correlated influenza-specific CD8^+^ T cells with protection against symptomatic pandemic influenza (5). Based on their protective role, CD8^+^ T cells are an attractive target in vaccine development. But, it is of note that CD8^+^ T cells only function after cellular infection has occurred, acting to limit rather than prevent infection. Understanding the protection provided by vaccine-induced CD8^+^ T cells in the context of virus-specific antibody is important in designing new influenza vaccines.

One approach to induce different types of adaptive immune response is to direct antigen to specific antigen-presenting cells (6). This can be achieved using dimeric vaccines with targeting modules (either a scFv derived from an antibody or chemokine) coupled by a dimerization unit to the antigen (7). Changing the module allows the targeting of different antigen-presenting cells, leading to different types of immune responses and enabling the comparison of the relative contribution of different adaptive immune effectors. In previous studies in mice, an MHC-targeting module has been shown to induce a dominant IgG1 antibody response with some T cell induction (8), while an XCL1 chemokine module led to a more skewed CD8^+^ T cell response (9).

In the current study, we investigated the relative contribution of influenza-specific CD8^+^ T cells induced by a DNA vaccine in a heterologous prime-boost regime with a protein vaccine. The protein vaccine induced a strong antibody response but relatively few CD8 cells. Induction of CD8^+^ T cells by the dimeric vaccine improved the resolution of disease, and when CD8 cells were depleted, the enhanced resolution was no longer observed. However, CD8 cells alone were insufficient to protect against infection. Based on this, we conclude that vaccine-induced CD8 responses are beneficial but are supplementary to antibody.

## Materials and Methods

### DNA Vaccine Constructs

The generation of the DNA vaccine constructs containing the targeting unit, the dimerization unit consisting of h1 + h4 + C_H_3 domains derived from human IgG3, and antigen has been previously described (9, 10). The constructs either expressed amino acids 18–541, the extracellular domain, and part of the transmembrane domain, of influenza A/California/07/2009 (H1N1) hemagglutinin or the conserved IYSTVASSL epitope of H1 (533–541) as the antigen payload, and anti-I-E^d^ MHC class II single chain variable fragment (scFv) from the 14-4-4S monoclonal antibody that binds the conserved E alpha chain, or murine XCL1 as the targeting unit. All sequences were synthesized by Eurofins MWG (Germany) or GenScript (USA). The synthesized inserts were subcloned into the expression vector pUMVC4a on *Not*I and *Bgl*II, all including either an Ig VH signal peptide or the murine XCL-1 signal peptide to ensure secretion. The αMHCII:HA (Cal/07) construct has been described previously (8).

### Mouse Immunization and Infection

Six- to eight-week-old female CB6F1, BALB/c, or C57BL/6 mice were obtained from Harlan UK Ltd. (Bradford, UK) and kept in specific pathogen-free conditions in accordance with the UK’s Home Office guidelines, and all work was approved by the Animal Welfare and Ethical Review Board (AWERB) at Imperial College London. Studies followed the ARRIVE guidelines. Animals were immunized in a prime (d0)-boost (d21)-challenge (d42) regime and culled on day 7 of challenge (d49 relative to prime). For protein immunization, mice were immunized intramuscularly (i.m.) with 0.1 μg purified surface antigens from influenza strain H1N1 A/California/7/2009 (GSK Vaccines, Siena, Italy) in 50 μl. For DNA vaccination, mice were injected i.m. into the *anterior tibialis* with 5 μg plasmid in 50 μl of sterile PBS followed by electroporation (EP). Two lots of 5 pulses of 150 V with switched polarity between pulses were delivered using a CUY21 EDIT system (BEX, Japan). For infections, mice were anesthetized using isoflurane and infected intranasally (i.n.) with 5 × 10^4^ PFU of influenza A H1N1 (strain A/England/195/2009). Where used, CD8^+^ T cells were depleted using two intraperitoneal injections of 0.25 mg anti-murine CD8 antibody clone YTS156, and CD4^+^ T cells were depleted with 0.125 mg each of YTA3 and YTS191 (a kind gift of S. Cobbold, Oxford University) on day −1 and +1 of infection (11).

### Influenza

H1N1 influenza (strain A/England/195/2009), isolated by Public Health England in the UK, April 2009 (12), was grown in Madin–Darby Canine Kidney (MDCK) cells, in serum-free DMEM supplemented with 1 μg/ml trypsin. The virus was harvested 3 days after inoculation and stored at −80°C. Viral titer was determined by plaque assay as previously described (13).

### Semiquantitative Antigen-Specific ELISA

Antibodies specific to influenza H1N1 were measured using a standardized ELISA (14). IgG responses were measured in sera and IgA responses in bronchoalveolar lavage. MaxiSorp 96-well plates (Nunc) were coated with 1 μg/ml H1N1 surface proteins or a combination of anti-murine lambda and kappa light chain-specific antibodies (AbDSerotec, Oxford, UK) and incubated overnight at 4°C. Plates were blocked with 1% BSA in PBS. Bound IgG was detected using HRP-conjugated goat anti-mouse IgG (AbD Serotec). Bound IgA was detected using a biotinylated anti-IgA and a streptavidin-HRP. A dilution series of recombinant murine IgG or IgA was used as a standard to quantify specific antibodies. TMB with H_2_SO_4_ as stop solution was used to detect the response and optical densities read at 450 nm.

### Tissue and Cell Recovery and Isolation

Mice were culled using 100 μl intraperitoneal pentobarbitone (20 mg dose, Pentoject, Animalcare Ltd., UK) and tissues collected as previously described (15). Blood was collected from carotid vessels and sera isolated after clotting by centrifugation. Lungs were removed and homogenized by passage through 100 μm cell strainers, then centrifuged at 200 × *g* for 5 min. Supernatants were removed, and the cell pellet treated with red blood cell lysis buffer (ACK; 0.15M ammonium chloride, 1M potassium hydrogen carbonate, and 0.01 mM EDTA, pH 7.2) before centrifugation at 200 × *g* for 5 min. The remaining cells were resuspended in RPMI 1640 medium with 10% fetal calf serum and viable cell numbers determined by trypan blue exclusion.

### Influenza Viral Load

Viral load *in vivo* was assessed by Trizol extraction of RNA from frozen lung tissue disrupted in a TissueLyzer (Qiagen, Manchester, UK). RNA was converted into cDNA, and quantitative RT-PCR was carried out using bulk viral RNA, for the influenza M gene and mRNA using 0.1 μM forward primer (5′-AAGACAAGACCAATYCTGTCACCTCT-3′), 0.1 μM reverse primer (5′-TCTACGYTGCAGTCCYCGCT-3′), and 0.2 μM probe (5′-FAM-TYACGCTCACCGTGCCCAGTG-TAMRA-3′) on a Stratagene Mx3005p (Agilent technologies, Santa Clara, CA, USA). M-specific RNA copy number was determined using an influenza M gene standard plasmid.

### Flow Cytometry

Live cells were suspended in Fc block (Anti-CD16/32, BD) in PBS-1% BSA and stained with surface antibodies: influenza A H1 HA_533–541_ IYSTVASSL Pentamer R-PE (Proimmune, Oxford, UK), CD3-FITC (BD, Oxford UK), CD4-APC (BD), and CD8-APC Alexa75 (Invitrogen, Paisley, UK). Analysis was performed on an LSRFortessa flow cytometer (BD). FMO controls were used for surface stains.

### Statistical Analysis

Calculations as described in figure legends were performed using Prism 6 (GraphPad Software Inc., La Jolla, CA, USA).

## Results

### Heterologous Prime-Boost Regimes Using Dimeric DNA Vaccines Induce Both Antibody and CD8^+^ T Cell Responses and Improve Resolution of Disease

Vaccine-induced, antibody-mediated protection against influenza is well characterized, but CD8^+^ T cells are also important. DNA vaccines allow the induction of strong cellular responses (16), and the use of different targeting modules allows us to compare the relative contributions of different effectors (17). We compared the response to immunization using a DNA vaccine encoding dimeric APC-targeted antigen alone or in combination with protein antigens. The DNA vaccine construct for these studies encoded the HA gene from influenza Eng/195 (H1N1) dimerized to an anti-I-E^d^ MHC class II scFv with a dimerization unit consisting of h1 + h4 + C_H_3 domains from human IgG3. CB6F1 mice were used for these studies, and they are the F1 cross of BALB/c (I-E^d^) and C57BL/6 (I-E^b^) strains. Mice were immunized once with 5 μg DNA encoding the dimeric vaccine construct (αMHCII:HA) i.m. with EP, with or without a boost (on day 21), using a sub-protective dose of H1N1 proteins (0.1 μg) from CAL/09. Three weeks after the boost immunization (on day 42), mice were challenged i.n. with H1N1 influenza (strain A/England/195/2009) and culled 7 days later (day 49).

**Figure 1:**
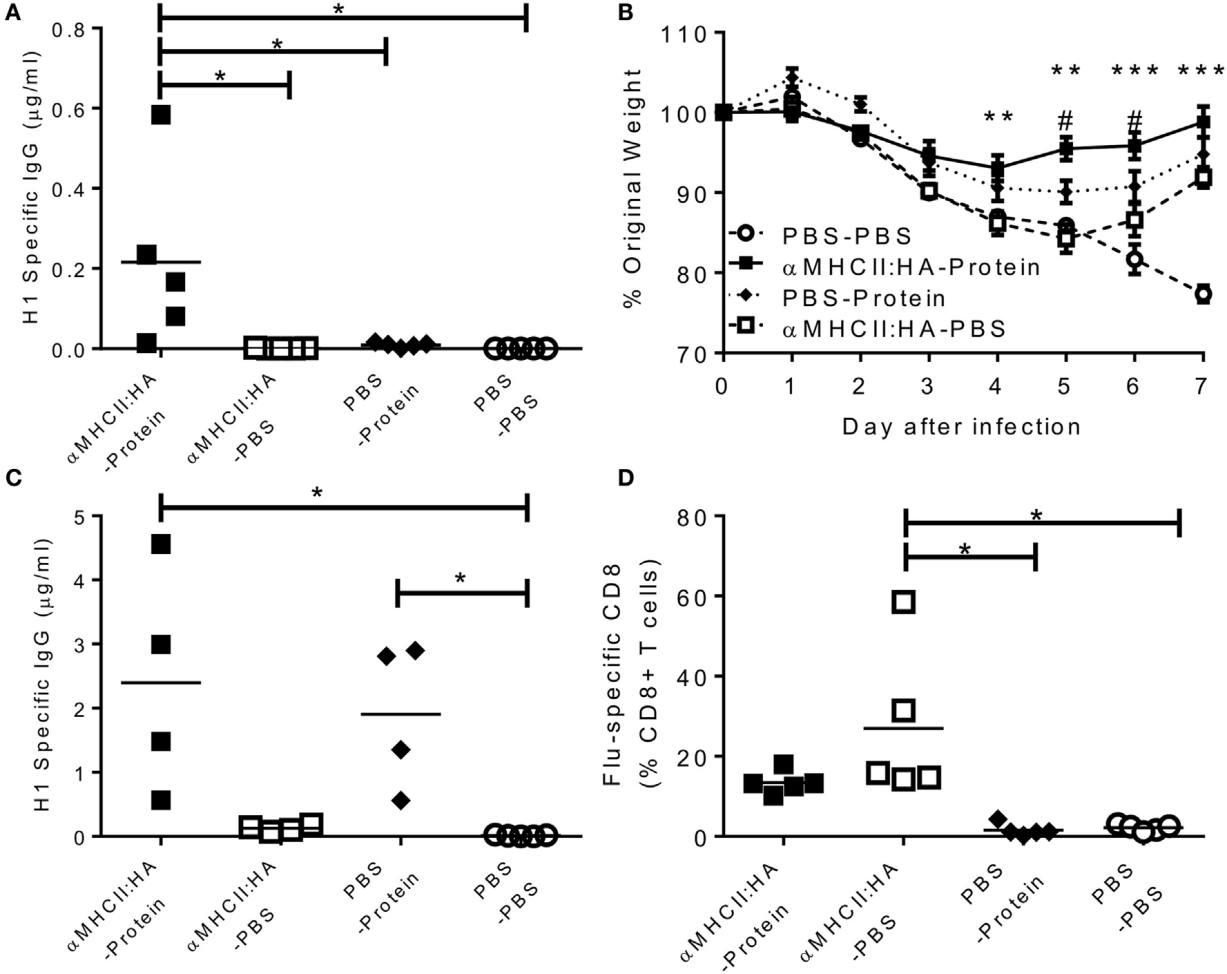
**Heterologous prime-boost regimes using dimeric DNA vaccines induce both antibody and CD8^+^ T cell responses and improves resolution of disease**. Mice were immunized intramuscularly (i.m.), immediately followed by electroporation, with 5 μg DNA encoding an MHCII-targeting:HA construct or 0.1 μg H1N1 proteins or DNA and then protein. Animals were infected intranasally (i.n.) with 5 × 10^4^ PFU A/England/195/2009 H1N1 influenza. One day before infection, IgG was assessed in sera **(A)**. Weight change was measured after infection **(B)**. H1 Influenza-specific IgG by ELISA **(C)** and influenza-specific CD8^+^ T cells **(D)** were measured on day 7 after infection. Lines and points represent mean of *n* ≥ 4 mice **p < 0.01, ***p < 0.001 between MHCII:HA-Protein and MHCII:HA, ^#^*p* < 0.05 between MHCII:HA and protein alone measured by one way **(B,C)** or two-way ANOVA **(A)**.

Blood was collected prior to infection to determine anti-influenza antibodies. αMHCII:HA primed-protein boosted animals had significantly more antibody than protein alone or αMHCII:HA alone (*p* < 0.05, Figure 1A). All immunizations gave some reduction of weight loss following influenza infection. The αMHCII:HA alone group recovered faster on days 6 and 7 after infection than PBS control mice, and a similar phenotype was seen after immunization with protein alone. However, prime immunization with DNA and then protein boost led to significantly improved recovery from d4 after infection (*p* < 0.05 compared to DNA or protein alone on d5 and d6, Figure 1B). After infection, antibody responses in the αMHCII:HA-Protein group were the same as the PBS-protein group, and levels were 10-fold higher than before infection (Figure 1C). There was some detectable antibody after immunizing with αMHCII:HA alone, which was slightly boosted by infection. However, αMHCII:HA alone immunized animals had a significant influenza-specific CD8^+^ T cell response in the lungs, as measured by pentamer-positive cells, greater than the protein alone or naive animals (*p* < 0.05, Figure 1D). These cells were also induced in the prime-boost group. These data suggest that while antibody is protective against influenza infection, antigen-specific CD8^+^ T cells contribute to recovery in the absence or near absence of antibodies.

### Accelerated Resolution in Prime-Boost Regimes Is Provided by CD8^+^ T Cells

**Figure 2:**
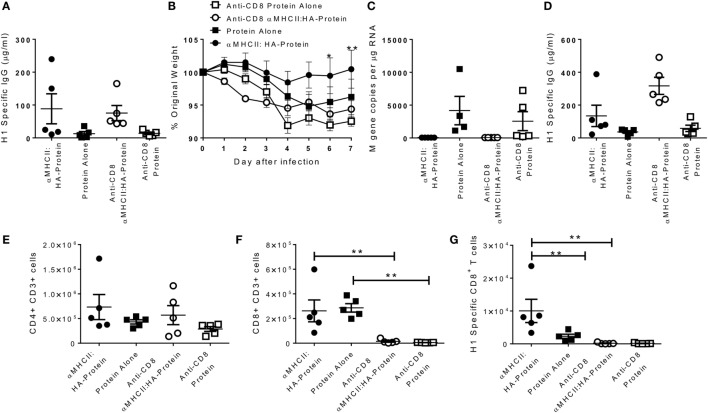
**CD8^+^ T cells required for accelerated resolution of DNA prime-boost regimes**. CB6F1 mice were immunized i.m. with 5 μg DNA encoding MHCII:HA, then 0.1 μg H1N1 proteins, or protein alone. 21 days later, mice were infected intranasally with H1N1 influenza. CD8^+^ T cells were depleted by antibody (YTS156) on day −1 and +1 of infection. One day before infection, IgG was assessed in sera **(A)**. Weight change was measured after infection **(B)**. M gene copy number **(C)**, H1 Influenza-specific antibody by ELISA **(D)**, CD4^+^ **(E)**, and CD8^+^ **(F)** and influenza-specific CD8^+^ T cells **(G)** were measured on day 7 after infection. Lines and points represent mean of *n* = 5 mice *p < 0.05, **p < 0.01 between MHCII:HA-protein and MHCII:HA-protein αCD8 measured by one-way ANOVA.

Having observed that heterologous prime-boost immunization led to faster recovery, and the DNA vaccines induced both an influenza-specific CD8 and antibody response, we wished to determine the role of the CD8 cells. Mice were immunized with αMHCII:HA with a protein boost or protein alone, and responses compared between animals treated with CD8 depleting antibody and control during infection. As seen before, αMHCII:HA-Protein immunization induced more antibody than protein alone 21 days after the boost immunization (Figure 2A). αMHCII:HA-Protein-immunized, CD8^+^-depleted mice lost significantly more weight than the immunized animals with intact CD8^+^ responses (*p* < 0.05 on day 6 and 7, Figure 2B). CD8 depletion had no effect on protein alone immunization. At day 7 after infection, αMHCII:HA-Protein-immunized mice had no detectable viral load, and CD8 depletion had no effect on this (Figure 2C). CD8 depletion also had no effect on the antibody response (Figure 2D) or CD4^+^ T cell number in the lungs (Figure 2E) but led to a significant reduction in both total (Figure 2F) and influenza-specific CD8^+^ T cells (Figure 2G). From this, we conclude that the improved recovery seen after αMHCII:HA priming before protein vaccination is partially mediated by CD8^+^ cells.

### CD8^+^ Cells in Isolation Are Not Sufficient for Protection from Influenza Infection

**Figure 3:**
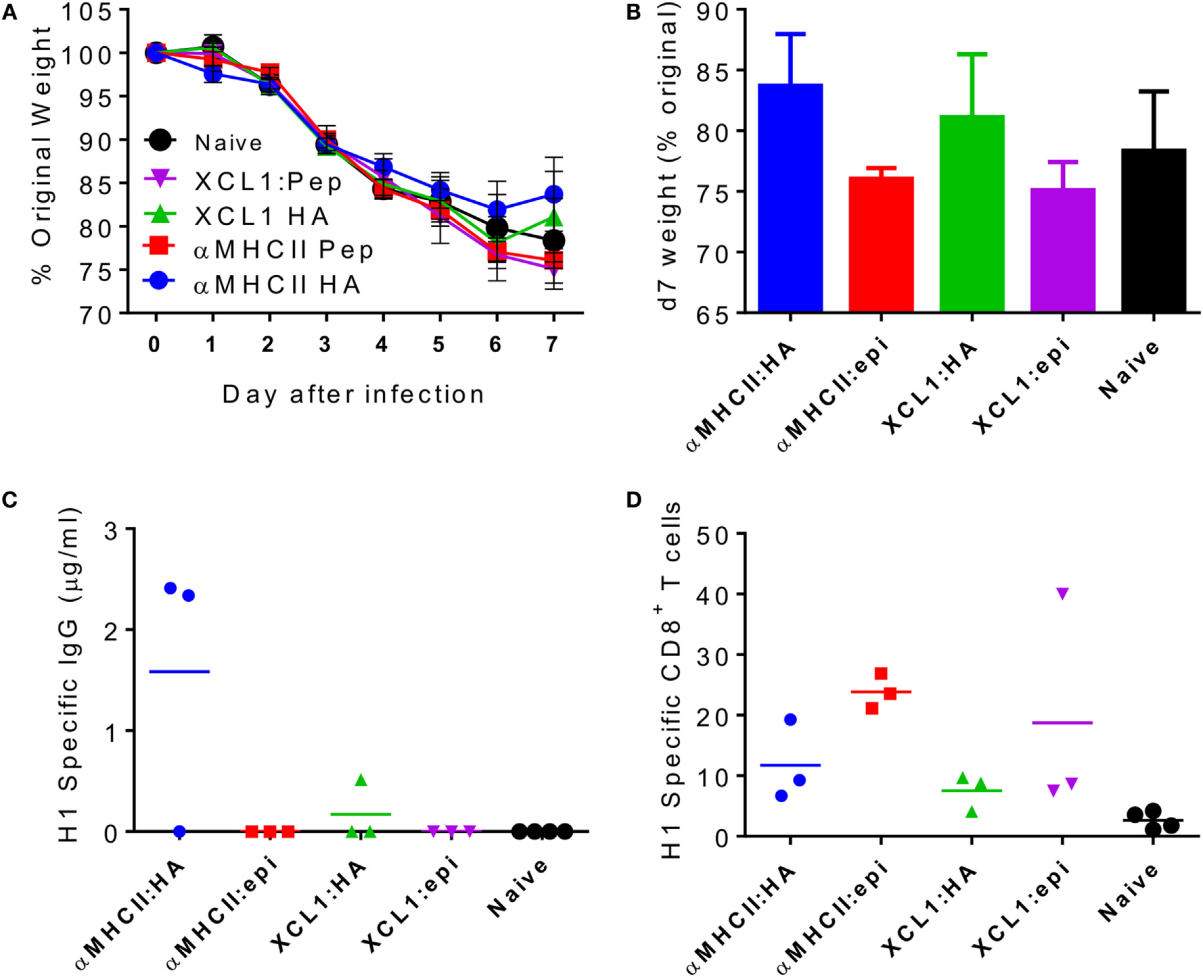
**Pilot study comparing CD8 response to different dimeric vaccine constructs**. CB6F1 mice were primed i.m. with 5 μg DNA constructs targeting either MHC II or the XCR1 chemokine receptor, conjugated to the full HA peptide or the immunodominant epitope alone (epi). Animals were infected i.n. with Eng/195 H1N1 influenza. Weight change was measured after infection **(A)**, with day 7 weight alone shown for clarity **(B)**. H1 Influenza-specific antibody by ELISA **(C)** and influenza-specific CD8 T cells **(D)** were measured on day 7 after infection. Lines and points represent mean of *n* ≥ 3 mice.

Since we observed that CD8 cells contribute to the accelerated resolution of disease in the prime-boost immunization, we wished to determine whether vaccines inducing influenza-specific CD8 alone could also improve disease resolution. A pilot study was performed to determine the immune response vaccine constructs using different targeting unit/antigen combinations, in order to select the ones that gave the greatest CD8^+^ T cell responses. Mice were immunized with constructs encoding either anti-I-E^d^ scFv or the XCL1-targeting module with either the full HA surface domain (of Cal/07) or the K^d^ immunodominant epitope alone in H1 hemagglutinin (HA_533–541_ IYSTVASSL). The groups immunized with constructs encoding the epitope alone were not protected against influenza infection (Figures 3A,B). The more complete HA constructs offered modest protection with αMHCII:HA-immunized animals recovering slightly faster than the naive animals, and the XCL1:HA-immunized animals gaining weight on d7 post infection. There were striking differences in the antibody responses: only animals immunized with a construct expressing the whole HA had detectable antibody responses, and the response to the MHCII-targeting construct was greater than the XCL1 (Figure 3C). While the antibody responses were poor to these constructs, there was substantial recruitment of influenza-specific CD8^+^ T cells. All immunized groups had influenza-specific T cells in the lungs, but there were greater responses in the epitope-immunized animals (Figure 3D). From this pilot study, we conclude that the epitope-only vaccines induce a stronger CD8 response.

**Figure 4:**
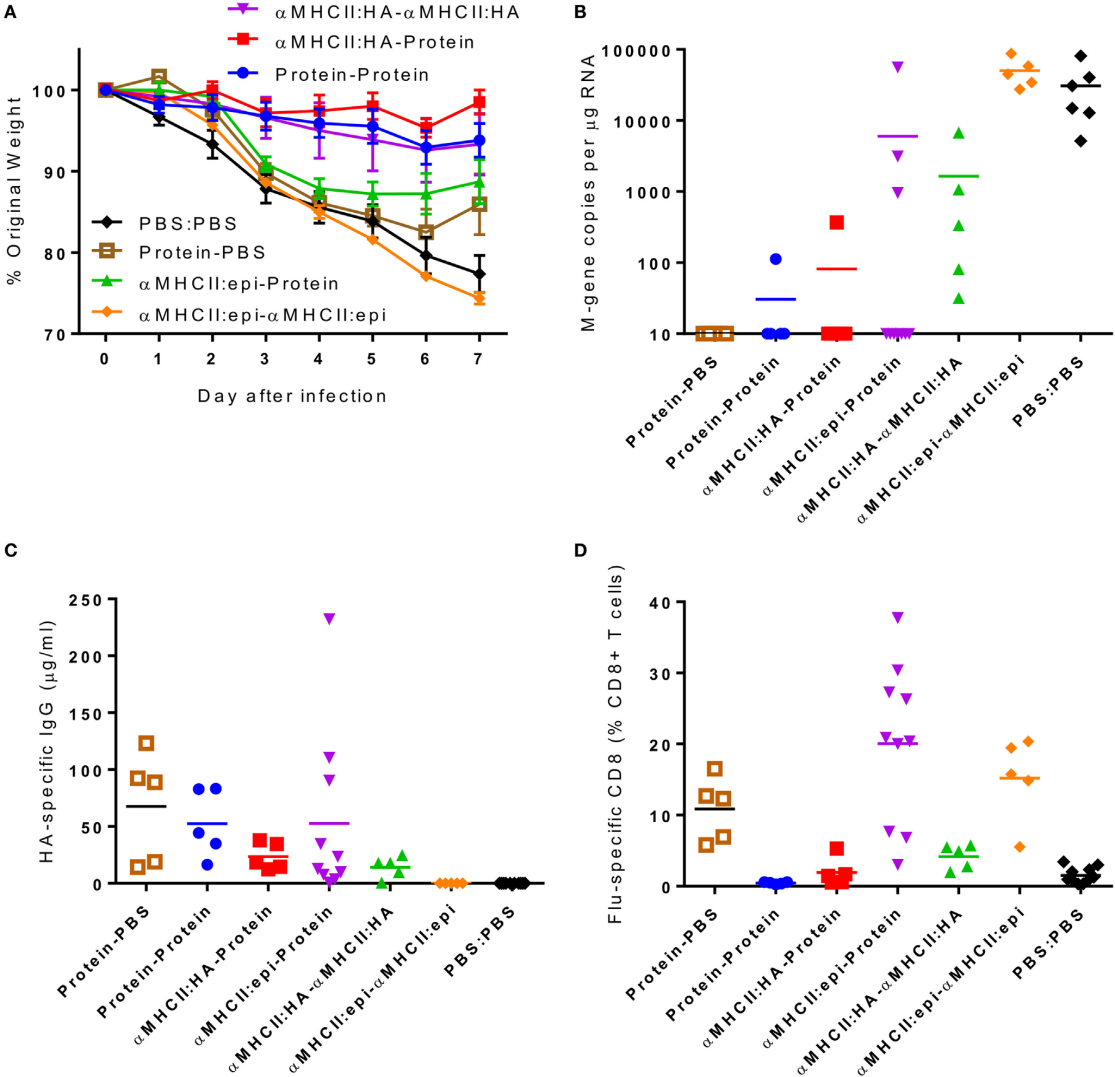
**The combination of antigens used in the prime-boost regime is critical in determining outcome**. CB6F1 mice were primed i.m. with 5 μg DNA constructs encoding MHCII:HA, or MHCII:Epitope, or 0.1 μg HA1 protein prior either heterologous or homologous boost. Twenty-one days after boost, animals were infected i.n. with Eng/195 H1N1 influenza. Weight change was measured after infection **(A)**. M gene copy number **(B)**, H1 influenza-specific antibody by ELISA **(C)**, and influenza-specific CD8^+^ T cells **(D)** were measured on day 7 after infection. Lines and points represent mean of *n* = 5 mice *p < 0.05, **p < 0.01 measured by one-way ANOVA.

To assess the relative contributions of CD8^+^ cells versus antibody, we took advantage of the differential responses to the αMHCII:HA or αMHCII:Epitope constructs, with either a DNA or protein boost, prior to infection with influenza. Prime-boost regimes with protein or αMHCII:HA (Eng/195) led to significant protection against infection, with little difference between the homologous or heterologous prime-boost regimes in weight loss (Figure 4A). Protein-containing regimes (Protein–Protein or αMHCII:HA-Protein) had slightly less detectable viral RNA in the lungs than the αMHCII:HA homologous regime (Figure 4B). The groups receiving a protein vaccination had more antibody than the other groups (Figure 4C), though it was surprising that there was no boost in antibody response after the second protein immunization. The regimes using the αMHCII:Epitope induced the greatest level of CD8^+^ cells in the lungs after infection (*p* < 0.05, Figure 4D), but the αMHCII:Epitope-immunized animals were not protected against infection, losing a similar amount of weight as naive animals and having an equivalent viral load. Priming with αMHCII:Epitope followed by protein did lead to significantly more CD8^+^ T cells than Protein–Protein but had little effect on protection. As seen before, the protein-only immunization regime did not induce any influenza-specific CD8^+^ T cells. These data suggest influenza-specific CD8^+^T cells targeting the IYSTVASSL epitope of H1 are not sufficient to protect against infection.

### Mouse Strain Key Determinant of Protection for MHCII Targeting Constructs

**Figure 5:**
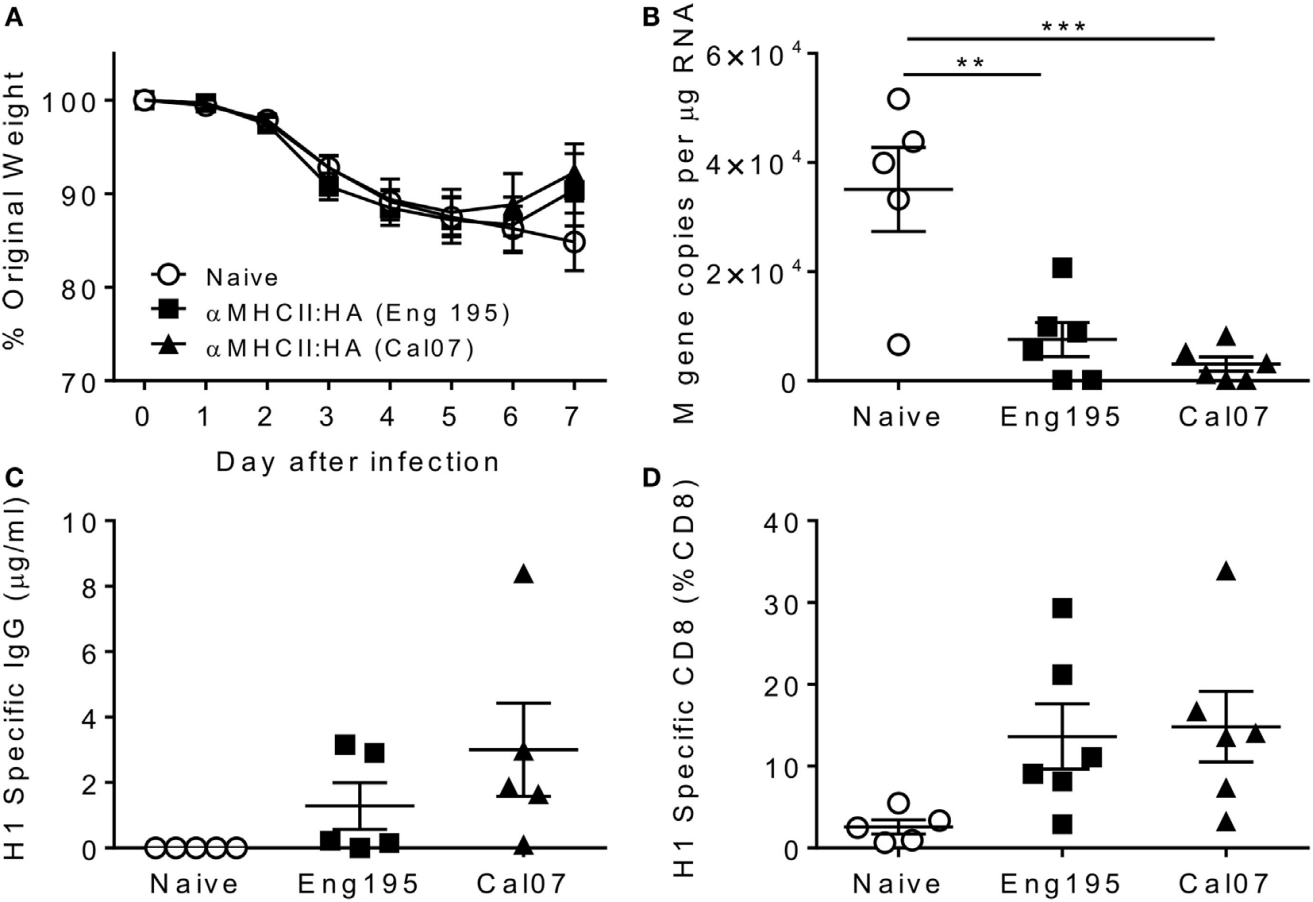
**Incomplete protection with both Cal07- and Eng195-encoding constructs in CB6F1 mice**. Mice were immunized intramuscularly with 5 μg DNA encoding different antigen-targeting module constructs with electroporation, prior to infection intranasally with 5 × 10^4^ PFU A/England/195/2009 H1N1 influenza. Weight change was measured after infection **(A)**. M gene copy number **(B)**, H1 Influenza-specific antibody by ELISA **(C)**, and influenza-specific CD8^+^ T cells **(D)** were measured on day 7 after infection. *n* = 5 animals per group, **p < 0.01 and ***p < 0.001 using ANOVA and posttest.

In previous studies using similar DNA vaccine constructs in BALB/c mice, complete protection against Cal/07 infection was observed after a single DNA vaccination (8). Possible sources of differences include the amount of DNA delivered (25 μg in published, 5 μg in current), the route of delivery (i.d. in published, i.m. in current), viruses used for challenge (Cal/07 in published, Eng/195 in current: the HA genes from Cal/07 and Eng/195 are 99% identical, with 4 amino acid changes), the mouse strains used (BALB/c in published, CB6F1 in current), or the antigens inserted into the MHCII-targeted construct. To ensure that there was no difference between constructs used in the current study and the published constructs, we compared immunization with the construct used in the previous study (8) and a construct expressing the HA from Eng/195. CB6F1 mice were immunized with 5 μg of each construct with EP, and 28 days later, they were infected i.n. with 5 × 10^4^ PFU of ENG195. Weight was measured daily after infection, and there was no difference between mice immunized with the two vaccine constructs; immunized mice recovered faster than naive mice on day 7 after infection (Figure 5A). Significantly, more viral RNA was detected in the lungs of previously naive animals than in immunized animals, and there was no difference in viral load between mice immunized with either construct (Figure 5B). Both constructs induced an immune response, as there was detectable specific IgG in the sera at d7 (Figure 5C) and flu-specific CD8^+^ T cells in the lung (Figure 5D) in immunized but not naive animals. From this, we conclude that the incomplete protection observed in the initial studies was not due to the construct, the antigen targeted, or the challenge virus, suggesting that mouse strain may be important, though the dose and route may also contribute to differences seen.

### Compatibility of Host Strain and Vaccine Construct MHC-Targeting Unit Is Critical in Level of Protection

**Figure 6:**
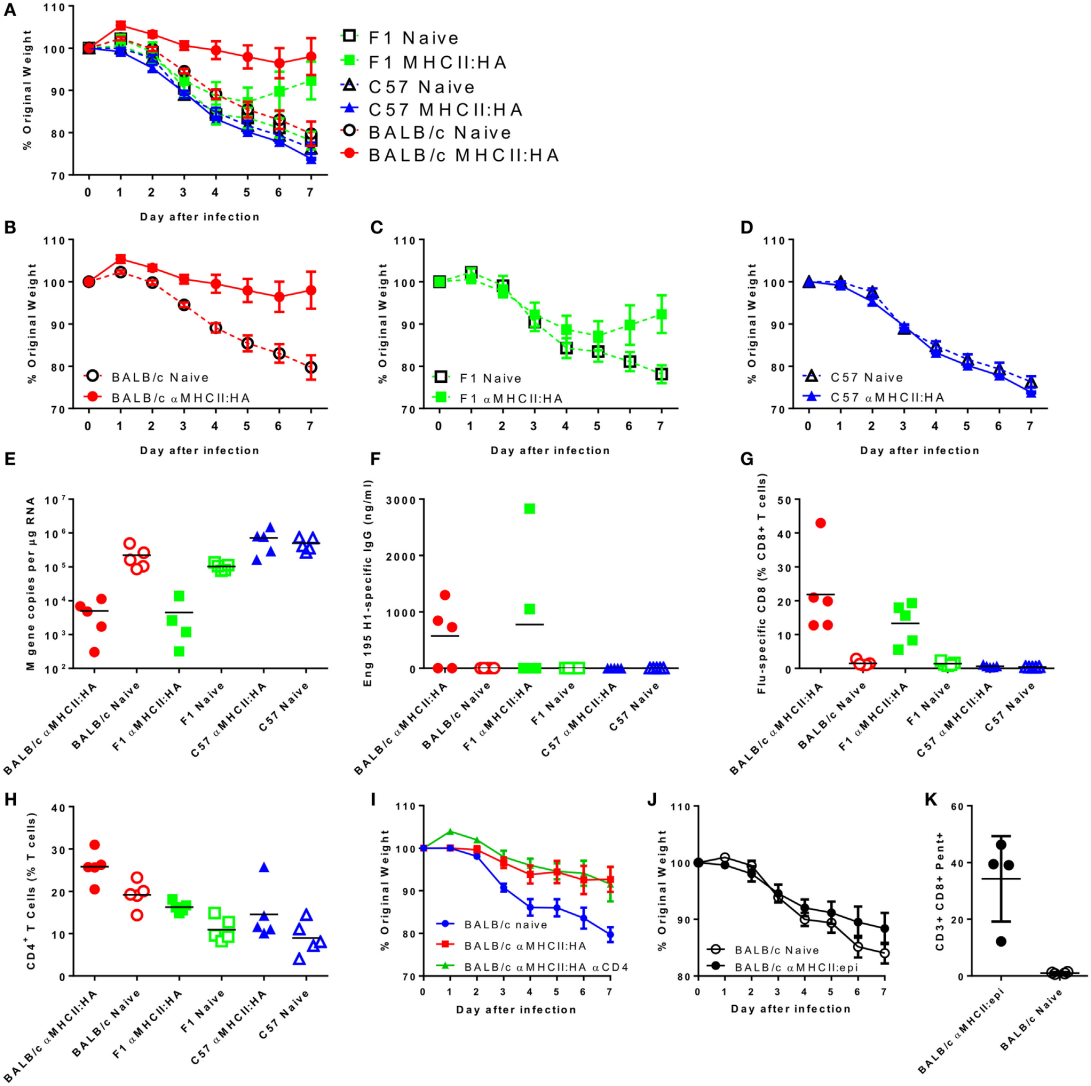
**Strain compatibility with MHC-targeting module affects protection against challenge**. Mice were immunized intramuscularly with 5 μg DNA encoding an I-E^d^ MHCII targeting-HA construct with electroporation, prior to infection intranasally with 5 × 10^4^ PFU A/England/195/2009 H1N1 influenza. Weight change was measured after infection **(A)**. The same data are presented by strain for clarity: BALB/c **(B)**, CB6F1 **(C)**, and C57BL/6 **(D)**. M gene copy number **(E)**, H1 Influenza-specific antibody by ELISA **(F)**, influenza-specific CD8^+^ T cells **(G)**, and % CD4^+^ T cells **(H)** were measured on day 7 after infection. Weight loss of MHCII:HA immunized BALB/c mice treated with CD4 depleting antibody during infection **(I)**. BALB/c mice were immunized two times intramuscularly with 5 μg DNA encoding an I-E^d^ MHCII targeting-epitope construct with electroporation prior to infection. Weight change was measured after infection **(J)**. On day 7 after infection, percentages of influenza-specific CD8^+^ T cells were quantified **(K)**. *n* = 5 animals per group.

The targeting unit of the MHC vaccine construct is based on an scFv, derived from the 14-4-4S monoclonal antibody that binds the conserved E alpha chain of the I-E^d^ MHCII molecule, which is expressed in mouse strains that are H-2^d^. We have previously observed that mouse strain is critical in the recall immune response to respiratory viral infection (18). Previously published studies with similar MHC-targeting vaccine constructs used BALB/c mice (H-2^d^), and the current studies used CB6F1 mice, which are mixed H-2^d^ and H-2^b^. To test whether mouse strain has an effect on the immune response to the vaccine, we immunized BALB/c (H-2^d^), C57BL/6 (H-2^b^), and CB6F1 (mixed H-2^d^ and H-2^b^) with the αMHCII:HA construct. These animals were then challenged with influenza. Naive animals started losing weight on day 2 after infection, and this weight loss continued to day 7, at which point the animals were culled (Figure 6A). There was no significant difference in the magnitude or the profile of the weight loss between the naive animals regardless of strain, indicating that baseline susceptibility to influenza was similar. However, there was a striking difference in protection based on MHC genotype. BALB/c were more protected than F1 mice, which were more protected than the C57BL/6 mice, directly reflecting the amount of I-E^d^ MHC (Figures 6B–D) and reflecting the previously published study (8). Likewise, there was only a reduction in viral load in the BALB/c and F1-immunized mice (Figure 6E). There was detectable influenza-specific antibody (Figure 6F) and CD8^+^ T cells (Figure 6G) in both the BALB/c and CB6F1 mice, and there was no difference between the two strains, suggesting that there are other components that contribute to protection against infection. The BALB/c mice had a higher proportion of CD4^+^ T cells in the lungs, which may have contributed to protection (Figure 6H), but in a separate study when treated with CD4-depleting antibody during challenge, there was no effect on resolution of disease after depletion (Figure 6I).

In the CB6F1 mice, the regimes that induced CD8^+^ T cells alone did not protect against infection. Since we observed a difference between BALB/c and CB6F1 mice in protection following immunization with the αMHCII:HA construct, we wished to determine whether there was a difference in the protective capacity of the CD8^+^ T cells induced in H-2^b^ mice. BALB/c were immunized twice with the MHCII-epitope construct prior to infection with influenza. Mice were not protected against infection (Figure 6J) despite inducing an extremely high influenza-specific CD8 response (Figure 6K). As with the CB6F1 mice, no antibody response was seen after immunization with this construct (data not shown). These studies clearly demonstrate the effect of the targeting module on the response.

## Discussion

In this study we observed that a DNA vaccine encoding a dimeric construct that targets hemagglutinin to antigen-presenting cells can induce an influenza-specific CD8^+^ T cell response, which in the context of antibody can lead to more rapid recovery from infection. CD8 cell depletion removed the extra protection provided by the DNA vaccination. It should be noted that the MHCII:HA DNA-prime protein-boost regime induced more antibody than protein alone prior to infection, which will contribute to the additional protection seen; but the depletion studies suggest that the additional protection provided by elevated antibody was secondary to that provided by CD8. From this, we conclude that CD8 contribute to protection against influenza infection but are insufficient when acting alone.

The H1 hemagglutinin epitope (IYSTVASSL) only DNA constructs were insufficient to protect against influenza infection in spite of inducing robust CD8 responses in the lung during infection. There were a number of possible reasons why immunization that only induces a CD8 response fails to protect against influenza challenge including immunopathology, the infectious dose used, the DNA vaccine dose used, targeting a poorly protective epitope or immunizing the wrong tissue. Excess CD8^+^ T cells can be associated with disease, both for influenza (19) and respiratory syncytial virus (RSV) infection (20). But, there was little evidence for CD8 cells causing enhanced immunopathology in the current study – for example, the MHCII:Epitope construct induced little antibody and high levels of CD8 cells, but the disease profile was the same as naive mice. A different epitope might be more protective; the current study evaluated a CD8 epitope in hemaglutinin, and CD8 responses against the NP protein of influenza have been explored for vaccine candidates (21). Though in other studies, the IYSTVASSL (HA533) epitope has been used as a heterologous boost vaccine, expressed by *Listeria* (22), leading to heterosubtypic immunity, differences in route, dose, regime, and vector of delivery could all contribute to the differences seen. It is possible that, in the absence of antibody, CD8 cells are being swamped by virus: with a smaller infectious challenge dose, CD8 cells may have provided more protection (23), but the viral dose used has been carefully titrated to give a clear disease phenotype. It was of note that the αMHCII:HA and other DNA vaccines used in other studies (24, 25) were protective against the same dose of the same virus; notably, all of these regimes induced antibody and CD8 T cells, indicating that both are required.

Another possibility is that the CD8 cells induced by vaccination were in the wrong tissue. Systemic vaccination is most likely to lead to systemic T cell memory, resident in the spleen, whereas CD8 in the lung are required to clear the infection. The time taken to recruit cells in response to infection from the systemic to the local compartment may account for the failure to clear the infection. Tissue-resident memory CD8 cells have been shown to be critical in protection, and vaccine regimes that induce them have a significantly improved outcome compared with systemic vaccination (26). One approach might be to use live viral vaccines - the use of the live attenuated influenza vaccine led to the induction of influenza-specific CD8 T cells in the lungs (27) - and we have recently shown that heterologous prime-boost strategies including viral vectors can alter the immune outcome (28).

One of the striking observations was the effect of mouse MHC genotype on the response to the MHC-targeting vaccine constructs. This shows that the targeting component of the dimeric vaccine is critical in the response it induces. But the homozygous I-E^d^ strain (BALB/c) was most protected against infection, in spite of having broadly similar antibody and CD8^+^ T cell responses to the heterozygous CB6F1 mice. C57BL/6 mice were not protected due to their lack of I-E^d^. One possibility is that hemagglutinin-specific CD4 cells were also induced by the vaccine and there more of these in the BALB/c mice than the CB6F1. There are two well-characterized MHCII epitopes in H1-derived hemagglutinin SVSSFERFEIFPK (H2-IE^d^ positions 124–136) and HNTNGVTAACSHE (H2-IA^d^ positions 139–151), and αMHCII:HA can induce responses against these (8). The role of CD4 cells in protection against influenza is less well characterized than CD8, but recent studies have shown a correlation between CD4 T cell responses and protection in a human influenza challenge study (29). Whether these cells play a role and what role they play – either as helpers (30) or as cytotoxic T cells (31) – is not clear, though the depletion of CD4 during challenge had no effect on disease outcome, suggesting that they are not acting as cytotoxic effectors but may be important in priming the response.

The best protection was observed when both CD8 and antibody were induced. This reflects other studies using the dimeric vaccine constructs expressing hemagglutinin (8, 9, 32) all of which induced both antibody and CD8 T cells. We believe that CD8 play a critical role in the later stages of the infection leading to viral clearance and recovery from influenza infection, providing an adjunct to antibody-mediated protection. Studies in human RSV showed that the probability of protection from antibody follows a sigmoidal distribution suggesting a role for other factors (33). We propose a model where antibody prevents the initial colonization, but if antibody is evaded by the virus, then CD8 cells enable more rapid clearance. In this context, vaccines that can induce local CD8 responses may be of value, particularly if they target conserved epitopes.

## Author Contributions

LL, EK, JM, and JT performed studies; GG, ES, and ML developed and provided reagents; GG, BB, AF, and JT wrote paper; JT and AF designed studies.

## Conflict of Interest Statement

ES, ML, and AF are employed by Vaccibody, which generated the constructs. BB, AF, and GG are inventors on patent applications filed on the vaccine molecules by the TTO offices of the University of Oslo and Oslo University Hospital, according to institutional rules. BB is head of the Scientific panel, and AF is CSO of Vaccibody AS. They both hold shares in the company. The remaining authors declare that the research was conducted in the absence of any commercial or financial relationships that could be construed as a potential conflict of interest.
